# The association between dysglycemia and neonatal respiratory distress syndrome severity as well as short-term clinical outcomes at hospital admission

**DOI:** 10.3389/fped.2026.1772116

**Published:** 2026-04-17

**Authors:** Fang Liu, Xiaochun Li, Yan Lu, Fangqi Hu

**Affiliations:** Department of Pediatrics, Anqing Municipal Hospital, AnQing, AnHui, China

**Keywords:** blood glucose, disease severity, hyperglycemia, neonatal respiratory distress syndrome, short-term outcomes

## Abstract

**Objective:**

To evaluate the impact of admission blood glucose levels on disease severity and short-term outcomes in neonates with neonatal respiratory distress syndrome (NRDS).

**Methods:**

This retrospective cohort study included 219 NRDS neonates admitted to a tertiary hospital in China (August 2022–April 2024). Participants were categorized into hypoglycemia (*n* = 38), normoglycemia (*n* = 143), and hyperglycemia (*n* = 38) groups based on initial whole-blood glucose. Associations between glucose levels, NRDS severity, and clinical outcomes were analyzed.

**Results:**

The incidence of severe NRDS was significantly higher in the hyperglycemia group compared to the normoglycemia group (56.4% vs. 38.7%, *P* = 0.019). Hyperglycemia positively correlated with prolonged mechanical ventilation, oxygen therapy, and elevated lactate levels (all *P* < 0.05). Neonates with hyperglycemia also exhibited higher rates of specific complications, including intracranial hemorrhage (ICH) and bronchopulmonary dysplasia (BPD), whereas those with hypoglycemia were more susceptible to hypoxic-ischemic encephalopathy and necrotizing enterocolitis. Separate multivariate logistic regression analyses revealed distinct predictive patterns: admission hyperglycemia exhibited a strong inverse (negative) association with arterial pH (OR = 0.000; 95% CI: 0.000–0.009) and a direct (positive) association with WBC count (OR = 1.086; 95% CI: 1.025–1.150). Conversely, admission hypoglycemia demonstrated an inverse association with pulmonary surfactant use (OR = 0.327; 95% CI: 0.124–0.859) and a direct positive association with patent ductus arteriosus (OR = 3.775; 95% CI: 1.441–9.890).

**Conclusion:**

Admission hyperglycemia predicts severe NRDS, prolonged respiratory support, and distinct complication profiles. Both hypoglycemia and hyperglycemia demonstrate specific independent predictors linked to metabolic disturbances and increased treatment demands. These findings highlight the clinical relevance of early glucose monitoring, providing new insights that admission dysglycemia may serve as an early biomarker of disease severity to guide risk stratification and personalized management of NRDS.

## Introduction

Neonatal respiratory distress syndrome (NRDS) is a severe respiratory disorder characterized by alveolar-capillary membrane injury and pulmonary surfactant deficiency (1, 2). It clinically manifests as progressive respiratory distress and represents one of the most common respiratory morbidities in neonates, especially preterm infants (3). Due to its rapid progression, delayed intervention often leads to high mortality and poor prognosis (4). The onset of NRDS is driven by multiple perinatal factors, among which prematurity, elective cesarean section, and perinatal asphyxia are significant risks (5).

Maternal gestational diabetes mellitus (GDM) is a major perinatal complication closely linked to NRDS, primarily because maternal hyperglycemia can severely impair fetal pulmonary surfactant synthesis and maturation (6). Elevated HbA1c levels during early pregnancy significantly increase the risk of adverse neonatal outcomes, particularly NRDS (7), with offspring of GDM mothers exhibiting a 1.5-fold higher risk of developing the syndrome (8). Furthermore, maternal GDM profoundly alters neonatal blood glucose homeostasis (9), frequently predisposing these infants to early adverse outcomes such as hypoglycemia (10). Crucially, neonatal dysglycemia itself has a significant correlation with the occurrence and progression of NRDS (11). In scenarios involving perinatal asphyxia, enhanced anaerobic glycolysis rapidly induces hypoglycemia (12), which, when compounded by existing glucose metabolism disorders, drastically elevates the risk of severe cerebral injury and mortality (13).

Despite these established pathophysiological connections, current literature predominantly focuses on the risk factors for NRDS onset (6). In most clinical research, neonatal blood glucose is treated merely as a secondary consequence rather than being evaluated as an independent, early predictor of disease severity (14). This limitation is partly due to methodological inconsistencies, including variable sampling timings and a lack of standardized diagnostic thresholds across studies (15). Additionally, many existing studies rely on cross-sectional or retrospective designs that fail to clearly establish predictive relationships between early admission glucose levels and subsequent disease outcomes (16).

To address these specific gaps, this study aims to systematically investigate the impact of admission blood glucose levels and dysglycemia on NRDS severity and short-term clinical outcomes. By evaluating early glucose status, we seek to establish a reliable biomarker that facilitates risk stratification and optimizes targeted early interventions, ultimately aiming to slow disease progression and improve short-term prognoses in affected neonates.

## Materials and methods

### Study population and inclusion/exclusion criteria

This retrospective cohort study included 219 neonates diagnosed with NRDS at Anqing Municipal Hospital, China (August 2022–April 2024). Inclusion criteria comprised postnatal age <24 h and absence of feeding before blood sampling. Exclusion criteria encompassed congenital anomalies (e.g., airway/cardiac malformations), inherited. This retrospective study was approved by the Institutional Review Board of Anqing Municipal Hospital, China (Approval No. 2025-32). All participating pregnant women were fully informed and provided written informed consent.

As this was a retrospective observational study utilizing pre-existing clinical data, an *a priori* sample size calculation was not performed. Instead, to ensure the robustness and representativeness of the findings, we included all consecutive eligible neonates admitted to our center during the predefined study period (August 2022–April 2024). This consecutive sampling strategy was employed to maximize the available sample size and statistical power while minimizing potential selection bias, thereby comprehensively reflecting real-world clinical scenarios.

### Blood glucose classification

Currently, while neonatal glucose cut-offs can vary in the literature depending on clinical objectives, the thresholds used in this study were based on widely recognized diagnostic criteria and operational definitions (14, 17). Neonatal blood glucose levels measured within 24 h after birth (before initial feeding) were categorized into three groups. The Hypoglycemia group was defined as a whole-blood glucose level <2.2 mmol/L (<40 mg/dL) (*n* = 38; 50.0% male; Gestational age 33.52 ± 1.93 weeks). This cut-off is an internationally recognized operational threshold for intervention and risk assessment in the first 24 h of life, supported by the American Academy of Pediatrics (AAP) and classic neonatal guidelines (14, 18). The Hyperglycemia group was defined as a whole-blood glucose level >7.0 mmol/L (>126 mg/dL) (*n* = 38; 60.5% male; Gestational age 34.36 ± 2.64 weeks). In neonatology, hyperglycemia is classically defined as a whole-blood glucose concentration >125 mg/dL (6.9 mmol/L) or a serum/plasma glucose concentration >150 mg/dL (8.3 mmol/L) (17, 19). Because our study utilized an ABL90 blood gas analyzer, which inherently measures whole-blood glucose, the >7.0 mmol/L threshold is methodologically accurate. Furthermore, although some treatment-oriented guidelines suggest higher thresholds (8–10 mmol/L) for initiating pharmacological interventions like insulin therapy, we specifically selected >7.0 mmol/L based on its pathophysiological relevance to NRDS. During acute respiratory distress, severe stress and inflammation trigger marked catecholamine release and insulin resistance. Substantial evidence indicates that even mild stress-induced hyperglycemia (>7.0 mmol/L) can precipitate osmotic diuresis, hemodynamic instability, impaired lung fluid clearance, and pulmonary surfactant dysfunction, thereby increasing the risk of adverse outcomes such as intraventricular hemorrhage and prolonged respiratory support well before glucose levels reach 8–10 mmol/L (19, 20). Thus, this cut-off serves as an early biomarker of disease severity and short-term prognosis rather than a strict trigger for medical intervention. The Normoglycemia group included neonates with glucose levels between 2.2 and 7.0 mmol/L (*n* = 143; 60.8% male; Gestational age 33.25 ± 2.81 weeks). NRDS diagnosis, severity assessment, and treatment adhered to *Practical Neonatology* (21) and *European Consensus Guidelines (*5).

### Initial glucose sampling and analysis

Immediately upon admission to the neonatal intensive care unit (NICU), arterial blood gas analysis and hematological testing were performed on all neonates. It is crucial to note that all initial blood samples were obtained strictly prior to the initiation of enteral feeding and before the administration of any intravenous fluids, dextrose infusions, maternal/neonatal corticosteroids, or other medications that could potentially alter glucose metabolism. Peripheral arterial blood samples (0.5 mL) were collected under aseptic conditions, transferred into lithium heparin-anticoagulated tubes, and analyzed within 5 min using a Siemens RAPID Point 500 automated blood gas analyzer (National Medical Device Registration No. 20172225057; Model ABL90). To ensure diagnostic accuracy and rule out pre-analytical errors, any abnormal glucose value (hypoglycemia <2.2 mmol/L or hyperglycemia >7.0 mmol/L) detected via the initial blood gas analysis was immediately verified by a confirmatory peripheral capillary blood glucose measurement. Subsequent diagnostic evaluations included bedside chest radiography, cranial ultrasonography, echocardiography, and abdominal ultrasonography. All procedures were rigorously standardized and executed in compliance with institutional Standard Operating Protocols.

### Clinical data collection

Demographic and clinical data were systematically recorded, including Sex, birth weight, Apgar scores, gestational age; Maternal age, gestational diabetes status, Arterial blood gas analysis, complete blood count, and biochemical parameters; Use of mechanical ventilation, pulmonary surfactant (PS) administration, and length of hospital stay; Patent ductus arteriosus (PDA), retinopathy of prematurity, bronchopulmonary dysplasia (BPD), hypoxic-ischemic encephalopathy (HIE), et al. Furthermore, recognizing the well-established impact of maternal gestational diabetes mellitus (GDM) on neonatal respiratory and metabolic outcomes, neonates born to diabetic mothers were explicitly included in the study cohort. To rigorously control for this potential confounder, maternal GDM status was systematically recorded as a baseline demographic variable and statistically compared across the three blood glucose groups to ensure baseline comparability prior to evaluating the independent effects of neonatal admission dysglycemia.

The diagnosis of NRDS was established based on classic clinical manifestations appearing shortly after birth—such as progressive respiratory distress, tachypnea, expiratory grunting, nasal flaring, and cyanosis—combined with characteristic radiological findings (5). The severity of NRDS was subsequently graded based on the initial bedside chest radiograph obtained within 24 h of admission, in combination with these clinical manifestations. Radiographic findings were classified into four progressive grades: Grade I is characterized by diffusely decreased lung radiolucency with fine granular and reticular shadows; Grade II presents as Grade I progression with visible air bronchograms extending to the middle and outer lung fields; Grade III is marked by further decreased radiolucency with blurred cardiac and diaphragmatic borders; and Grade IV presents as ‘white lungs’ with complete obliteration of the cardiac and diaphragmatic borders. Based on these criteria, neonates were categorized into two severity cohorts. The mild-to-moderate group included infants with Grade I or II radiographic findings presenting with mild clinical symptoms such as grunting and dyspnea. The severe group comprised infants with Grade III or IV radiographic findings accompanied by severe clinical manifestations, including expiratory grunting, tachypnea after airway clearance, cyanosis, and recurrent apnea.

### Statistical methods

Statistical analyses were performed using SPSS 29.0. Categorical variables were expressed as frequencies (percentages) and compared using the chi-square (*χ*^2^) test or Fisher's exact test, as appropriate. For *post hoc* pairwise comparisons of categorical variables, z-tests with Bonferroni correction were applied. Furthermore, Odds Ratios (ORs) and 95% Confidence Intervals (CIs) were calculated to quantify the effect sizes and relative risks for key clinical complications. Continuous variables were first tested for normality. Normally distributed data were presented as mean ± standard deviation (SD) and analyzed using one-way analysis of variance (ANOVA); *post hoc* pairwise comparisons were performed using the Least Significant Difference (LSD) t-test. Skewed data were expressed as median (Q1, Q3) and analyzed using the Kruskal–Wallis H test, followed by Dunn's *post hoc* test for pairwise comparisons. To identify independent factors associated with distinct dysglycemic states, variables demonstrating significance (*P* < 0.05) in univariate analyses were incorporated into two separate multivariate binary logistic regression models (stepwise method): one evaluating admission hyperglycemia and another evaluating admission hypoglycemia, both referencing the normoglycemia group. A two-tailed *P*-value <0.05 defined statistical significance.

## Results

### Baseline characteristics across groups stratified by blood glucose levels

No significant differences (*P* > 0.05) were observed in baseline characteristics between neonates with different admission blood glucose levels. Specifically, the median birth weight was comparable among the hypoglycemia, normoglycemia, and hyperglycemia groups [2.05 (1.64, 2.59) vs. 1.91 (1.51, 2.58) vs. 2.21 (1.92, 2.71) kg, *P* = 0.076], as was the mean gestational age (33.52 ± 1.93 vs. 33.25 ± 2.81 vs. 34.36 ± 2.64 weeks, *P* = 0.073). Furthermore, there were no statistical differences in the proportions of male neonates (50.0% vs. 60.8% vs. 60.5%, *P* = 0.471) or the incidence of maternal gestational diabetes mellitus (26.3% vs. 21.0% vs. 15.8%, *P* = 0.530). Other clinical and demographic variables, including maternal age, 1-minute Apgar scores, birth asphyxia, primiparity, cesarean delivery, and multiple gestations, also demonstrated no significant variations across the three cohorts. Detailed comparisons are presented in Table 1.

**Table 1:** Analysis of the general situation of different blood glucose levels in neonates at admission [M (Q1, Q3), $\bar{X}\;$ ± SD, *N* (%)].

Variables	Hypoglycemia group (*N* = 38)	Normal glycemic group (*N* = 143)	Hyperglycemic group (*N* = 38)	*P* value
General situation				
Birth weight (Kg)	2.05 (1.64, 2.59)	1.91 (1.51, 2.58)	2.21 (1.92, 2.71)	0.076
Maternal age (Y)	32.00 (28.75, 35.25)	32.00 (28.00, 35.00)	32.00 (29.75, 36.00)	0.628
1 min Apgar (points)	8.00 (7.00, 8.25)	8.00 (7.00, 9.00)	8.00 (7.00, 9.00)	0.127
Gestational age (W)	33.52 ± 1.93	33.25 ± 2.81	34.36 ± 2.64	0.073
Gender [*N* (%)]				0.471
Male	19 (50.0)	87 (60.8)	23 (60.5)	
Female	19 (50.0)	56 (39.2)	15 (39.5)	
Birth asphyxia [*N* (%)]				0.555
Yes	15 (39.5)	54 (37.8)	11 (28.9)	
No	23 (60.5)	89 (62.2)	27 (71.1)	
Cesarean delivery [*N* (%)]				0.431
Yes	27 (71.1)	109 (76.2)	27 (71.1)	
No	11 (28.9)	34 (23.8)	11 (28.9)	
Multiple gestations [*N* (%)]				0.547
Yes	3 (7.9)	21 (14.7)	5 (13.2)	
No	35 (92.1)	122 (85.3)	33 (86.8)	
Amniotic fluid contamination [*N* (%)]				0.924
Yes	3 (7.9)	13 (9.1)	4 (10.5)	
No	35 (92.1)	130 (90.9)	34 (89.5)	
Maternal gestational diabetes [*N* (%)]				0.530
Yes	10 (26.3)	30 (21.0)	6 (15.8)	
No	28 (73.7)	113 (79.0)	32 (84.2)	
Primiparity [*N* (%)]				0.525
Yes	13 (34.2)	33 (23.1)	10 (26.3)	
No	28 (65.8)	110 (76.9)	28 (73.7)	

### Association of admission glucose levels with NRDS severity and respiratory-metabolic indicators

There were significant differences in the occurrence of severe NRDS among neonates with different admission blood glucose levels (*χ*^2^ = 7.947, *P* = 0.019). Specifically, neonates in the hyperglycemia group (56.40%) had a significantly higher risk of developing severe NRDS compared to the normoglycemia group (38.70%; OR = 2.05, 95% CI: 1.00–4.19) and the hypoglycemia group (23.70%; OR = 4.17, 95% CI: 1.58–11.00) (all *P* < 0.05). No significant difference was observed between the hypoglycemia and normoglycemia groups.

With the increase of blood glucose levels upon admission, the duration of non-invasive ventilation, mechanical ventilation, and oxygen inhalation all increased, as did Lactate (LAC) levels (H = 6.488, 12.954, 6.443, 6.513, respectively; all *P* < 0.05). *Post-hoc* pairwise comparisons revealed that the duration of mechanical ventilation and oxygen inhalation was the longest in the hyperglycemia group. Furthermore, the LAC levels in the hyperglycemia group [2.76 (2.24, 5.68) mmol/L] were significantly higher than those in the hypoglycemia group [2.41 (1.69, 3.67) mmol/L]. There were also statistically significant differences in platelet (PLT) count, white blood cell (WBC) count, and pH values among the three groups (H = 4.005, 12.279, 16.630, respectively; all *P* < 0.05). *Post-hoc* analyses revealed that neonates in the hyperglycemia group exhibited significantly lower pH values and higher WBC counts compared to both the hypoglycemia and normoglycemia groups. Additionally, the PLT count in the hyperglycemia group was significantly higher than that in the hypoglycemia group (Table 2).

**Table 2:** Analysis of the occurrence of severe NRDS and respiratory-metabolic indicators with different admission blood glucose levels.

Variables	Hypoglycemia group (*N* = 38)	Normal glycemic group (*N* = 143)	Hyperglycemic group (*N* = 38)	*χ*^2^/F/H	*P* value
PO2 (mmHg)	47.95 (40.00, 67.63)	46.70 (36.70, 67.00)	37.50 (28.43, 65.83)	4.035	0.133
PCO2 (mmHg)	52.00 (46.95, 59.00)	52.30 (45.70, 59.60)	53.05 (43.45, 59.00)	0.151	0.927
PH	7.23 (7.17, 7.27) ^a^	7.23 (7.16, 7.27) ^a^	7.16 (7.12, 7.21) ^b^	16.630	<0.001
LAC (mmol/L)	2.41 (1.69, 3.67) ^b^	2.80 (2.08, 3.79) ^ab^	2.76 (2.24, 5.68) ^a^	6.513	0.039
WBC (10^9/L)	11.42 (8.92, 15.63) ^b^	11.07 (8.14, 15.04) ^b^	14.54 (11.71, 20.04) ^a^	12.279	0.002
TBIL (uoml/L)	39.65 (32.90, 70.05)	44.50 (34.80, 68.70)	45.10 (38.30, 68.65)	1.153	0.562
ALT (IU/L)	5.00 (4.00, 8.00)	5.00 (4.00, 7.00)	6.00 (4.75, 8.25)	2.496	0.287
BUN (mmol/L)	3.60 (2.90, 5.00)	3.50 (2.70, 5.40)	3.50 (2.68, 4.70)	0.245	0.885
CREA (umol/L)	52.00 (37.75, 65.00)	53.00 (41.00, 70.00)	61.00 (46.75, 75.5)	3.699	0.157
CKMB (IU/L)	91.25 (51.83, 157.18)	77.00 (41.00, 140.70)	87.05 (45.15, 184.5)	1.233	0.540
PLT (109/L)	235.79 ± 61.12^b^	264.67 ± 62.61^ab^	271.16 ± 55.47^a^	4.005	0.02
Mechanical ventilation time (h)	0.00 (0.00, 0.00) ^c^	0.00 (0.00, 44.50) ^b^	1.50 (0.00, 82.25) ^a^	12.954	0.002
Noninvasive ventilation time (h)	90.50 (54.75, 140.5) ^b^	98.00 (69.50, 157.50) ^a^	80.00 (34.63, 115.5) ^b^	6.488	0.039
Oxygen inhalation time (h)	133.25 (69.75, 241.63) ^b^	140.00 (97.00, 120.00) ^b^	205 (111.25, 357.25) ^a^	6.113	0.047
Length of stay (d)	17.50 (12.00, 30.25)	16.00 (12.00, 31.00)	16.00 (14.00, 23.5)	0.416	0.812
NRDS condition [*N* (%)]				7.947	0.019
Severity	9 (23.7) ^b^	55 (38.7) ^b^	22 (56.4) ^a^		
Gently	29 (76.3)	87 (61.3)	17 (43.6)		

Post hoc pairwise comparisons were conducted among the three groups. Each superscript letter (a, b, c) denotes a subset of groups whose column proportions or medians do not differ significantly from each other. Specifically, groups sharing the same superscript letter are not significantly different (*P* > 0.05), whereas groups with completely different superscript letters demonstrate a statistically significant difference (*P* < 0.05). “ab” indicates no significant difference compared to either group “a” or group “b”.

### Association of admission glucose levels with NRDS complications and short-term clinical outcomes

Compared to the normoglycemia group, the overall risk of complications was significantly elevated in both the hyperglycemia group (OR = 7.28, 95% CI: 2.69–19.71) and the hypoglycemia group (OR = 2.12, 95% CI: 1.01–4.47). Furthermore, the hyperglycemia group presented a higher complication risk than the hypoglycemia group (OR = 3.43, 95% CI: 1.08–10.89) (all *P* < 0.05).

Neonates with hyperglycemia demonstrated a significantly increased risk for several specific interventions and adverse outcomes compared to the normoglycemia group, including the need for mechanical ventilation (OR = 2.16, 95% CI: 1.04–4.50), PS use (OR = 2.20, 95% CI: 1.03–4.69), PDA (OR = 2.09, 95% CI: 1.01–4.32), and intracranial hemorrhage (ICH) (OR = 2.26, 95% CI: 1.06–4.84). These risks were even more pronounced when comparing the hyperglycemia group to the hypoglycemia group (OR = 3.99, 3.32, 3.38, and 5.54, respectively; all *P* < 0.05). Additionally, the incidence of retinopathy of prematurity (ROP) was significantly higher in the hyperglycemia group (15.79%) than in the hypoglycemia group (0%) (*P* = 0.012). Regarding severe respiratory complications, the hyperglycemia group exhibited the highest incidence of pulmonary hemorrhage (10.50%) and BPD (10.50%), displaying significantly higher risks compared to the normoglycemia group (pulmonary hemorrhage: OR = 16.71, 95% CI: 1.81–154.29; BPD: OR = 8.29, 95% CI: 1.46–47.17) (all *P* < 0.05).

Conversely, the hypoglycemia group was particularly susceptible to a different cluster of complications, showing a significantly higher risk compared to the normoglycemia group for HIE (OR = 3.64, 95% CI: 1.15–11.58), NEC (OR = 10.68, 95% CI: 1.98–57.49), and PAH (OR = 3.05, 95% CI: 1.24–7.49). Other clinical parameters, including pneumothorax, gastrointestinal bleeding, PO2, PCO2, and length of hospital stay, showed no statistically significant differences among the three groups (*P* > 0.05). Detailed data are presented in Table 3.

**Table 3:** Analysis of the NRDS complications and short-term clinical outcomes with different admission blood glucose levels.

Variables	Hypoglycemia group (*N* = 38)	Normal glycemic group (*N* = 143)	Hyperglycemic group (*N* = 38)	χ^2^/F/H	*P* value
Complication [*N* (%)]				20.251	<0.001
Yes	25 (65.8) ^b^	68 (47.6) ^c^	33 (86.8) ^a^		
No	13 (34.2)	75 (52.4)	5 (13.2)		
Mechanical ventilation [*N* (%)]				7.788	0.02
Yes	7 (18.4) ^b^	42 (29.4) ^b^	18 (47.4) ^a^		
No	31 (81.6)	101 (70.6)	20 (52.6)		
Use of PS [*N* (%)]				6.738	0.034
Yes	15 (39.5) ^b^	71 (49.7) ^b^	16 (68.4) ^a^		
No	23 (60.5)	72 (50.3)	12 (31.6)		
PDA [*N* (%)]				6.492	0.039
Yes	8 (21.1) ^b^	43 (30.1) ^b^	18 (47.4) ^a^		
No	30 (78.9)	100 (69.9)	20 (52.6)		
PAH [*N* (%)]				6.289	0.043
Yes	10 (26.3) ^a^	15 (10.5) ^b^	6 (15.8) ^ab^		
No	28 (73.7)	128 (89.5)	32 (84.2)		
Pneumorrhagia [*N* (%)]				8.478	0.008
Yes	1 (2.6) ^ab^	1 (0.7) ^b^	4 (10.5) ^a^		
No	37 (97.4)	142 (99.3)	34 (89.5)		
Pneumothorax [*N* (%)]				3.706	0.169
Yes	2 (5.3)	9 (6.3)	6 (15.8)		
No	36 (94.7)	134 (93.7)	32 (84.2)		
ICH [*N* (%)]				9.103	0.011
Yes	4 (10.5) ^b^	32 (22.4) ^b^	15 (39.5) ^a^		
No	34 (89.5)	111 (77.6)	23 (60.5)		
HIE [*N* (%)]				6.210	0.045
Yes	6 (15.8) ^a^	7 (4.9) ^b^	5 (13.2) ^ab^		
No	32 (84.2)	136 (95.1)	33 (86.8)		
NEC [*N* (%)]				9.195	0.006
Yes	5 (13.2) ^a^	2 (1.4) ^b^	2 (5.3) ^ab^		
No	33 (86.8)	141 (98.6)	36 (94.7)		
Gastrointestinal hemorrhage [*N* (%)]				2.926	0.204
Yes	0 (0.0)	10 (7.0)	1 (2.6)		
No	38 (100.0)	133 (93.0)	37 (97.4)		
Retinopathy [*N* (%)]				11.196	0.003
Yes	0 (0.0) ^b^	3 (5.9) ^b^	6 (15.8) ^a^		
No	38 (100.0)	140 (97.9)	32 (84.2)		
BPD [*N* (%)]				6.485	0.023
Yes	1 (2.6) ^ab^	2 (1.4) ^b^	4 (10.5) ^a^		
No	37 (97.4)	141 (98.6)	34 (89.5)		

Post hoc pairwise comparisons were conducted among the three groups. Each superscript letter (a, b, c) denotes a subset of groups whose column proportions or medians do not differ significantly from each other. Specifically, groups sharing the same superscript letter are not significantly different (*P* > 0.05), whereas groups with completely different superscript letters demonstrate a statistically significant difference (*P* < 0.05). “ab” indicates no significant difference compared to either group “a” or group “b”.

### Multivariate analysis of indicators associated with admission hyperglycemia and hypoglycemia

To elucidate the independent clinical factors associated with different dysglycemic states, two separate multivariate binary logistic regression models (stepwise method) were constructed: one evaluating predictors of admission hyperglycemia (>7.0 mmol/L) and another for admission hypoglycemia (<2.2 mmol/L), both using the normoglycemia group as the reference (dependent variable: abnormal=1, normal=0).

Factors associated with admission hyperglycemia: The model identified that admission hyperglycemia was independently associated with pH, WBC count, and the occurrence of ICH. Specifically, arterial pH exhibited a strong inverse association (OR = 0.000, 95% CI: 0.000–0.009, *P* < 0.001), indicating that severe metabolic or respiratory acidosis drastically increases the likelihood of hyperglycemia. Conversely, an elevated WBC count demonstrated a positive association (OR = 1.086, 95% CI: 1.025–1.150, *P* = 0.005), suggesting that heightened systemic inflammation independently predicts hyperglycemia. The occurrence of ICH was also independently associated with the hyperglycemic state (OR = 0.353, 95% CI: 0.125–0.995, *P* = 0.049) (Table 4).

**Table 4:** Multivariate logistic regression analysis of indicators associated with admission hyperglycemia.

Variables	*β*	Wald χ^2^	*P* value	OR	95% CI
PH	−9.773	14.614	<0.001	0	0.000∼0.009
WBC	0.082	7.906	0.005	1.086	1.025∼1.150
ICH	−1.041	3.877	0.049	0.353	0.125∼0.995

Factors associated with admission hypoglycemia: The second model revealed that admission hypoglycemia was independently associated with WBC count, PLT count, duration of invasive mechanical ventilation, PS use, and the occurrence of PDA. Notably, the occurrence of PDA showed a strong positive association with hypoglycemia (OR = 3.775, 95% CI: 1.441–9.890, *P* = 0.007). In contrast, the use of PS demonstrated a significant inverse association (OR = 0.327, 95% CI: 0.124–0.859, *P* = 0.023), clearly distinguishing its clinical relationship from the hyperglycemic pattern. Other independent predictors included WBC count (OR = 1.089), PLT count (OR = 0.989), and mechanical ventilation time (OR = 0.977) (Table 5).

**Table 5:** Multivariate logistic regression analysis of indicators associated with admission hypoglycemia.

Variables	β	Wald χ^2^	*P* value	OR	95% CI
WBC	0.085	7.901	0.005	1.089	1.026∼1.155
PLT	−0.011	9.622	0.002	0.989	0.982∼0.996
Invasive mechanical ventilation time	−0.023	6.652	0.01	0.977	0.960∼0.994
Use of PS	−1.118	5.144	0.023	0.327	0.124∼0.859
PDA	1.328	7.309	0.007	3.775	1.441∼9.890

## Discussion

NRDS is a common and severe condition encountered in neonatal intensive care units, with a reported mortality rate as high as 24 percent. It may lead to serious long term neurological and respiratory sequelae (22, 23). Currently, there is a lack of effective early predictive biomarkers in clinical practice.

GDM is considered one of the major risk factors for NRDS in China (24). The current study found no significant association between neonatal blood glucose levels and maternal GDM, in contrast to findings from some previous studies (21, 22). Standardized prenatal monitoring and strict maternal glycemic control likely mitigate the direct impact on neonatal glucose metabolism. In addition, other potential confounding factors such as intrauterine fetal distress, stress responses during labor, or perinatal medical interventions may contribute to abnormal neonatal glucose levels (25). Despite this, monitoring neonatal blood glucose remains clinically important. Hyperglycemic neonates often present with more severe NRDS, which aligns with the findings reported by Salabai et al. (11). The underlying mechanisms may involve multiple pathophysiological processes. Hyperglycemia can suppress the secretion of thyroid hormones, which in turn reduces the expression of mRNAs related to pulmonary surfactant production, impairing fetal lung development and maturation (26), and ultimately increasing the risk of NRDS.

Neonatal glucose metabolism is highly vulnerable to disturbances due to the immature development of blood glucose regulatory mechanisms at birth. Dysglycemia profoundly impacts the clinical course. Severe hyperglycemia correlates with heightened systemic inflammation, severe acidosis, and prolonged respiratory support. Acute respiratory failure prevents adequate autonomous ventilation, leading to poor lung compliance (27). This creates a detrimental cycle where hypoxia and hypercapnia exacerbate blood gas acidosis and hyperlactatemia, further complicating glucose homeostasis. Persistent neonatal dysglycemia is notoriously difficult to correct and may induce cellular damage across muscle, cardiac, and neurological tissues, potentially leading to long term sequelae such as seizures and intellectual disability (28).

Abnormal blood glucose levels significantly elevate the incidence of short term complications (29). However, the target organs affected by hyperglycemia and hypoglycemia differ. Hyperglycemia predominantly drives vascular injury and inflammatory responses, predisposing neonates to PDA, pulmonary hemorrhage, ICH, ROP, and BPD. Elevated glucose induces oxidative stress, upregulates vascular endothelial growth factor expression, and inhibits insulin like growth factor 1, thereby disrupting normal angiogenesis (30). Additionally, hyperglycemia exacerbates hemodynamic instability and impairs circulatory perfusion (31, 32). In contrast, hypoglycemia is closely linked to ischemic and hypoxic damage, increasing susceptibility to HIE, NEC, and PAH. The neonatal brain is highly glucose dependent; therefore, severe energy deprivation rapidly inflicts irreversible neurological injury (33, 34). Hypoglycemia also aggravates ischemia and oxidative injury of the intestinal mucosa (35) and disrupts energy metabolism in the pulmonary vasculature, which can trigger abnormal remodeling and PAH (33).

Multivariate analysis further elucidates these divergent pathophysiological pathways. Severe acidosis acts as a strong independent predictor of hyperglycemia, likely reflecting the intensity of the acute stress response and subsequent catecholamine surge. Systemic inflammation, indicated by elevated WBC counts, independently associates with both dysglycemic states, highlighting severe inflammatory stress as a central destabilizer of metabolic homeostasis. Furthermore, the independent association between hypoglycemia and increased requirements for pulmonary surfactant and invasive mechanical ventilation underscores the profound energy depletion that accompanies severe respiratory failure.

**Figure 1:**
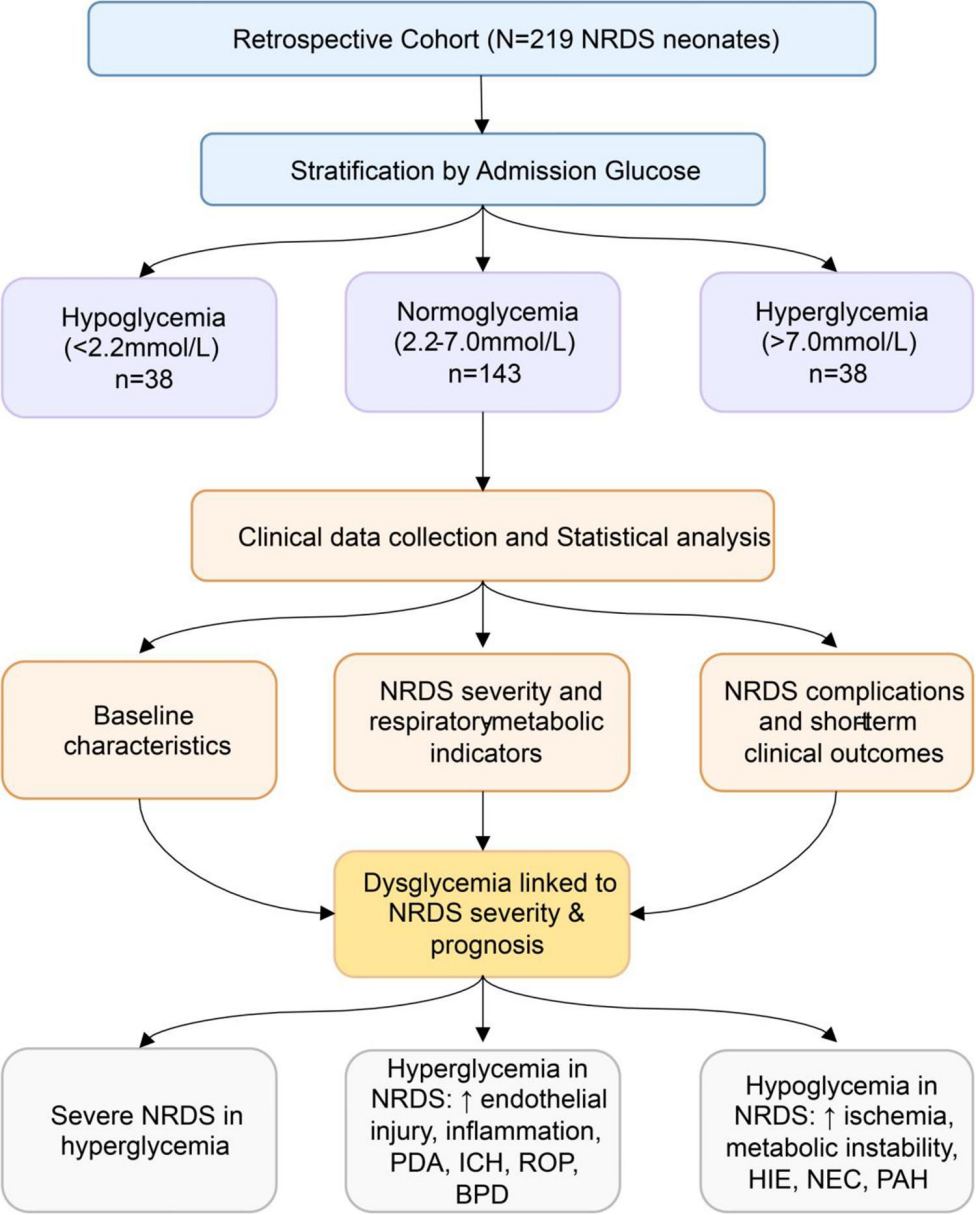
Study flowchart and conceptual model evaluating the impact of admission dysglycemia on NRDS severity and early clinical outcomes. The flowchart delineates the retrospective cohort stratification based on admission whole-blood glucose levels into hypoglycemia (<2.2 mmol/L), normoglycemia (2.2-7.0 mmol/L), and hyperglycemia (>7.0 mmol/L) groups. Following clinical data collection and statistical analysis, the conceptual model at the bottom summarizes the distinct pathophysiological trajectories and clinical outcomes independently associated with specific dysglycemic states. The upward arrow (↑) indicates an increase or exacerbation of the respective condition or pathological process. NRDS, neonatal respiratory distress syndrome; PDA, patent ductus arteriosus; ICH, intracranial hemorrhage; ROP, retinopathy of prematurity; BPD, bronchopulmonary dysplasia; HIE, hypoxic-ischemic encephalopathy; NEC, necrotizing enterocolitis; PAH, pulmonary artery hypertension.

In summary, admission dysglycemia serves as a crucial early biomarker reflecting the severity and multisystem burden of NRDS, as show in Figure 1. Early and systematic glucose monitoring should be a standard component of neonatal admission assessments. Implementing stratified interventions based on initial glucose levels could facilitate early risk identification and optimize personalized care. However, several limitations must be acknowledged. This single center retrospective study involved a limited sample size from eastern China, where regional disparities in perinatal care resources may limit external applicability (36). Additionally, the lack of continuous dynamic glucose monitoring prevents the assessment of longitudinal glycemic fluctuations, and unmeasured confounding factors cannot be entirely excluded. Future multicenter prospective studies integrating dynamic glucose profiles, insulin levels, and specific inflammatory markers are warranted to robustly validate these findings and elucidate the underlying pathogenesis.

## Data Availability

The raw data supporting the conclusions of this article will be made available by the authors, without undue reservation.
